# Gallbladder Polyps Are Associated with Proximal Colon Polyps

**DOI:** 10.1155/2019/9832482

**Published:** 2019-09-12

**Authors:** Kuan-Chieh Lee, Wen-Juei Jeng, Chen-Ming Hsu, Chia-Jung Kuo, Ming-Yao Su, Cheng-Tang Chiu

**Affiliations:** ^1^Department of Gastroenterology and Hepatology, Chang Gung Memorial Hospital at Linkou, Taoyuan, Taiwan; ^2^Liver Research Center, Division of Gastroenterology, Department of Gastroenterology and Hepatology, Chang Gung Memorial Hospital, Taoyuan, Taiwan

## Abstract

**Background:**

The association between gallbladder (GB) disease and colorectal precancerous lesions remains elusive. This study sought to explore the association between GB disease and colorectal neoplasms at different locations.

**Methods:**

Patients who received general health checkup from January to December 2008 were included and subgrouped into three groups by polyp location: proximal, distal, and whole colon. GB disease and other known risk factors for colon cancer were compared and analyzed. Different types of polyps at different locations were further investigated.

**Results:**

Of a total of 3136 patients (1776 men and 1360 women; mean age, 49.3 years) who had colon polyps, 212 (6.8%) had GB stone and 512 (16.3%) had GB polyps. Patients in the proximal colon polyp group had higher rates of GB polyps and stones. GB polyps were independently associated with proximal colon polyps, including both hyperplastic polyps (odds ratio, 1.523; *P* = 0.034) and adenomatous polyps (odds ratio, 1.351; *P* = 0.048). No relationship between GB polyps and distal or any colon polyps was observed. Irrespective of the polyp location (i.e., proximal, distal, or any part of the colon), GB stone did not show any association with colon polyp.

**Conclusions:**

We suggested that GB polyps are associated with proximal colon polyps. Colonoscopy may be a more effective strategy for screening proximal precancerous lesions among patients with GB polyps. The association between GB disease and colon polyps demands further prospective investigation.

## 1. Introduction

Colorectal cancer (CRC) is the fourth most commonly diagnosed cancer and the second leading cause of cancer-related deaths in the United States [1]. The 5-year survival rate for early-stage cancers is greater than 90%, whereas the 5-year survival rate for patients diagnosed with widespread cancer is less than 10% [2]. Some risk factors for CRC have been found to have site-specific characteristics. For example, a high-fiber diet was noted to reduce colon cancer risk [3], whereas processed red meat was associated with an increased risk of distal colon cancer [4]. Previous research has found differences in clinic-pathologic and prognostic features between proximal colon neoplasms and distal colon neoplasms [5–7]. In clinical practice, different manifestations have also been observed in tumors originating from different sites of the colon. Tumors originating from the proximal colon tend to present with insidious symptoms and signs, such as anemia and body weight loss, whereas tumors growing from the distal colon tend to present with local symptoms, such as changes in bowel habits and luminal obstruction [8–10]. Precancerous lesions at the proximal colon are poorly detected by both the fecal occult blood test and colonoscopy compared with those at the distal colon [11–13]. In addition, adenomatous polyps may be noted at proximal sites without the presence of distal adenoma [14, 15]. There are also many differences such as embryonic evolution, blood supply, lymphatic drainage, and lumen environment; thus, it is reasonable to subgroup colon cancer into proximal and distal groups according to tumor location rather than a single disease entity [16]. Because CRC can potentially be detected at early stages by screening through colonoscopy examinations in patients who have increased risk factors, identification of risk factors associated with colorectal polyps may facilitate screening and reduce CRC-related mortality [17–19].

The relationship between gallbladder (GB) disease and colorectal polyps has been of interest to many clinicians. Both these disease entities share some common risk factors [20–23]. Recently, a large cohort study of residents of Denmark linked gallstone disease to gastrointestinal tumors, especially for right-sided colon cancer, in the general population [24]. However, the pathophysiology and mechanism underlying this association are not well understood and fully explained. Because the temporal association has been based on ultrasound screen-detected gallstones and right-sided colon cancer, little is known regarding the causative behavior related to GB disease, including GB stones and GB polyps, for the occurrence of colon polyps in people around the world. Based on this concept, this study investigated whether GB disease is associated with colon polyps, especially of the proximal colon.

## 2. Materials and Methods

### 2.1. Study Participants and Design

**Figure 1:**
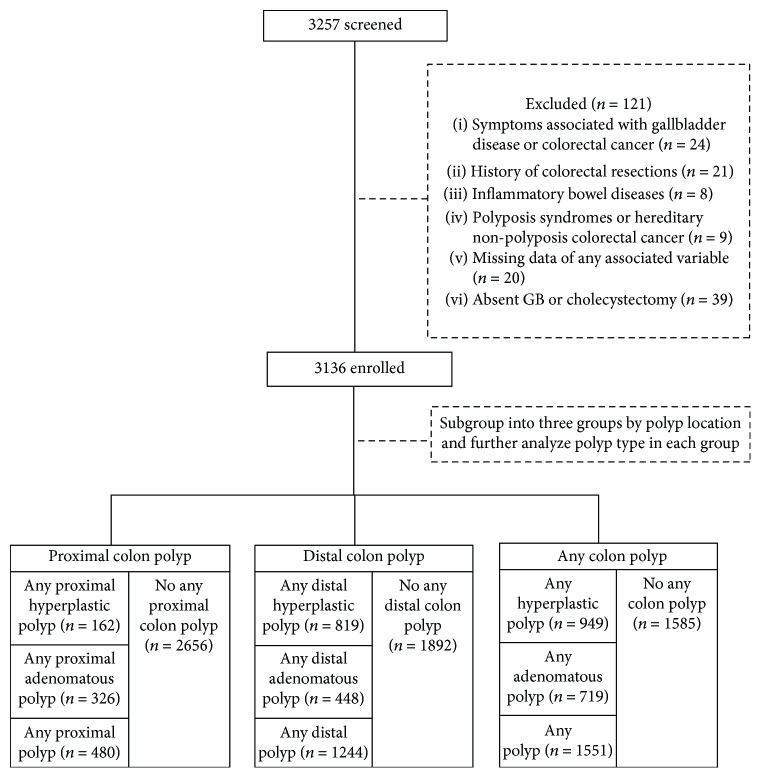
Flow chart of patients enrolled.

This cross-sectional study enrolled a total of 3273 patients who underwent a general health checkup, which included colonoscopy and abdominal sonography, between January 2008 and December 2008 at Chang Gung Memorial Hospital Taoyuan Branch. Demographic data, including age, sex, family history of CRC, and triglyceride and cholesterol levels, were collected in all enrolled patients. Patients with a history of colorectal resections, inflammatory bowel disease, polyposis syndromes or hereditary nonpolyposis CRC, missing data of any associated variable, and absent GB or previous cholecystectomy were excluded. A total of 121 patients were excluded (Figure 1). Considering that one patient may have more than one type of polyp located at different parts of the colon, we subgrouped our patients into three groups: proximal, distal, and whole colon. Each group was further subclassified and analyzed according to the polyp type by pathologic reports (Figure 1). All participants provided written informed consent. This study was conducted according to the principles expressed in the Declaration of Helsinki of the 1975 and was approved by the Chang Gung Medical Foundation Institutional Review Board (201701721B0D001).

### 2.2. Colonoscopy

All patients were instructed to take a colon preparation agent (either 2 L of polyethylene glycol (PEG) electrolyte solution or split-dose aqueous sodium phosphate solution) the day before the examination. The use of PEG for colon preparation was recommended when sodium phosphate was contraindicated. Patients underwent deep sedation with monitored anesthesia care during colonoscopy. An antispasmodic (10 mg of hyoscine methobromide, intravenously) was administered to patients with no contraindications. All colonoscopy programs were performed by experienced gastroenterologists by using standard video colonoscopy (CF260L, Olympus, Tokyo, Japan). The polyp size was determined by comparison to the size of an opened endoscopic forceps. The proximal and distal parts of the colon were divided by the splenic flexure. An advanced colorectal neoplasm was defined as the presence of a diameter of greater than 10 mm, high-grade dysplasia, or significant villous histology in more than 25% of its area [15]. We documented all polyps and recorded the size, location, and numbers of each in our report. The adenoma detection rate (ADR) among those older than 50 years was also calculated. All polyps were evaluated in accordance with the World Health Organization classification by experienced pathologists in our hospital [25].

### 2.3. Ultrasound

Abdominal ultrasonography was performed as a routine health checkup procedure after overnight fasting and prior colonoscopy. The liver, GB, pancreas, spleen, and kidneys were all examined. A GB polyp was diagnosed as a feature with hyperechoic immobile echo protruding from the GB wall into the lumen without acoustic shadowing, regardless of its histology. A GB stone was impressed as mobile with posture change and with acoustic shadowing [26].

### 2.4. Statistical Analysis

All data are presented as the mean ± standard deviation for continuous variables and as the number (percentage) of participants for categorical variables. Differences between categorical variables were analyzed using the chi-square test, and continuous variables were analyzed using Student's *t-*test. The nonparametric tests were applied where indicated. Logistic regression analysis was performed to determine the odds ratio (OR) and 95% confidence interval. Univariate analysis was applied for potentially relevant variables that differed between the two groups. Multivariate analysis was adjusted for those significant in the univariate analysis. Two-tailed *P* values less than 0.05 were considered statistically significant. All statistical analyses were performed using the Statistical Package for Social Science (SPSS package version 21, SPSS Inc., Chicago, IL, USA) for Windows.

## 3. Result

### 3.1. Demographic Characteristics of Study Patients

Of the total of 3136 patients, 1776 (56.6%) were men, 212 (6.8%) had GB stones, 512 (16.3%) had GB polyps, and their mean age was 49.3 years. The ADR among patients aged 50 years or older was 28%. Compared with patients who had no colon polyps, those with colon polyps were older, were predominantly male, and had a higher rate of GB stones (Table 1). In the proximal colon polyp group (480/1551, 31%), those who had any proximal colon polyp were older, were more likely to be male, and had a higher rate of GB stone, more GB polyps, and higher TG levels (Table 1). There was no significant baseline feature found in those who had any distal colon polyp (1244/1551, 80%) (Table 1).

### 3.2. Association between GB Disease and Colon Polyp

#### 3.2.1. Any Colon Polyp

The results of univariate and multivariate analyses showed that age, male sex, and family history of CRC were independently and positively associated any colon polyp (Table 2). An association with GB stones showed a statistical trend but without significance (*P* = 0.062). GB polyps did not show any significant association with any colon polyp. We also further analyzed data according to the polyp type. In the any hyperplastic polyp group, the results of univariate and multivariate analyses reported that those who had any hyperplastic polyp were older, predominantly male, and with a family history of CRC; those who had any adenomatous polyp were older and predominantly male. Both GB stones and GB polyps did not show any significant association with any hyperplastic or adenomatous polyps.

#### 3.2.2. Any Proximal Colon Polyp

The results of univariate and multivariate analyses revealed that older age, male sex, higher TG levels, and the presence of GB polyps (OR, 1.424; *P* = 0.006) were independently and positively associated with any proximal colon polyp (Table 3). GB stone did not show significance to any proximal colon polyp (OR, 1.174; *P* = 0.338). Regarding proximal hyperplastic polyp, parameter such as age, male subject, TG level, and GB polyp had positive association. Regarding proximal adenomatous polyp, only age, male subject, and GB polyp had positive association. GB stones were not found to be linked to any proximal hyperplastic and proximal adenomatous polyps.

#### 3.2.3. Any Distal Colon Polyp

The findings of univariate and multivariate analyses revealed that only family history of CRC was associated with distal hyperplastic polyps. No other significant variables were found to have associations with any distal colon polyp, including distal hyperplastic and adenomatous polyps (Table 4).

## 4. Discussion

In our study, we compared GB disease with other known risk factors to investigate the association of any independent risk factor with colon polyps. In addition, we classified and investigated different types of polyps at different locations. In contrast to previous research [27, 28], we found GB polyps to be positively associated with proximal colon polyps, including both hyperplastic and adenomatous polyps. This association was not found with GB stones; there was no relationship between GB polyps or GB stones with any distal colon polyps. To our knowledge, this is the first study to investigate the relationship between GB disease and different types of colon polyps located at different parts of the colon simultaneously in healthy individuals. However, a specific mechanism that explains our findings remains uncertain. The possible association between GB polyps and colorectal polyps can result from shared risk factors, and the development of both disease entities may be the consequence of the similar pathway involved [29, 30].

Since 1982, the association between GB disease, including gallstones, polyps, and postcholecystectomy status, and right-sided colon cancer has been suggested [31, 32]. Human studies have found a relationship between increased fecal secondary bile acids with colon polyps [33]. The predominance of cancers of the right side of the colon has been noted and has been explained as being caused by greater proximal colonic absorption of fecal secondary bile acids, which have been considered carcinogenic for many years [34]. However, a different mechanism is mentioned in other literatures that marked regional differences in bile acid metabolism between the right and left sides of the colon. Bile acids may lend a greater impact on the mucosa of the left side due to accumulation while passing through the colon [35]. Though a different point of view toward CRC, if an association between GB disease and proximal colorectal polyps could be proven by more research, then a low-cost, noninvasive examination, such as abdominal ultrasound, could be applied to identify individuals at risk of colorectal neoplasia.

One study conducted in southern Taiwan reported that both GB polyps and GB stones were associated with adenomatous polyps rather than other nonneoplastic polyps [36]. Our study, however, did not find an association between GB stones and adenomatous polyps in either the proximal or the distal colon. No marked differences were observed in baseline characteristics, such as age distribution, sex proportion, TG levels, and other significant confounding factors, between the two studies. This inconsistent result may be attributed to the difference in sample size and, more importantly, the documentation of the polyp type and polyp location; in the previous study, only the largest polyp was documented and recorded. In Korea, Hong et al. also found colorectal neoplasia to be significantly related to GB polyps, especially GB polyps larger than 5 mm [37]. Here, the possible cause of differing findings may be due to fewer male patients in our any adenomatous group and the association with hypertriglyceridemia being not significant in their multivariate analysis. One Western large cohort study found that GB stones are associated with right-sided colon cancer [24]; however, they did not examine whether existence of GB polyps had a similar impact in our study.

In all our patients, we did not find serrated polyps of the proximal colon in their pathology reports. This finding may be due to low awareness of the condition among gastroenterologists and poor communication with pathologists at that time. Additionally, there is poor interobserver agreement between pathologists in the differentiation of hyperplastic polyps from sessile serrated polyps [38]. Accumulating data suggest that serrated polyps may cause up to one-third of all sporadic CRC. Precancerous serrated polyps are predominately located in the right colon, which could explain why interval cancers most frequently appear in the proximal colon [39]. It is possible that there might be some serrated polyps among our enrolled patients. Because pathogenesis and clinical course of sessile serrated adenoma to adenocarcinoma are different from the traditional adenoma-carcinoma sequence [40], additional studies are needed to explore the relationship between GB disease and serrated polyps.

In our results, we found hypertriglyceridemia to be another strong significant risk factor for proximal colon polyps. Hypertriglyceridemia has been shown to modify bile acid excretion, circulating hormones, and energy supply to neoplastic cells [41]. Higher triglyceride levels can lead to a proinflammatory status within the body, leading to a proliferation of colorectal tumor cells [42]. The major impact on the proximal colon is possibly due to enterohepatic circulation of bile acid and TG, which may serve as risk factors for both disease entities. Additionally, hypertriglyceridemia only showed significance in the male population according to our subgroup analysis (data not shown). The effect of estrogen might play some protective role in this aspect.

GB polyps could be subcategorized as benign and neoplastic polyps. Cholesterol polyp (60–70%) is the most common benign GB polyps, whereas GB adenoma (1–5%) is the neoplastic polyps with malignant potential [43, 44]. In our study, the GB polyps are all based on abdominal ultrasonography without pathological proof, because patients enrolled did not accept cholecystectomy. Similar study about GB disease and CRC might be performed under pathological support for whom accepted cholecystectomy. Moreover, previous study had reported larger size of GB polyp was a predictor of GB cancer [43]; the effect between GB polyp size and CRC could be also further analyzed.

Growing attention has been given to the role of intestinal microbial infection in carcinogenesis [45]. In recent years, the relationship between intestinal microbiota and sporadic CRC has attracted much scientific interest. The composition and diversity of gut microbiota associated with CRC has been presented by many researchers [46, 47]. Enterotoxigenic *Bacteroides fragilis* and *Fusobacterium nucletum* are noted to be highly expressed in the CRC tissue compared with the matched tissue, and *F. nucletum* has been associated with high microsatellite instability [48]. Bile acid is also a mucosal protectant from toxigenic microbes that may invade through the luminal surface. In particular, among secondary bile acids, deoxycholic acid (DCA) has the most potent antimicrobial activity [49]. Additional studies are needed to explore the relationship between bile acids and microbiota in different parts of the colon.

The strengths of this study are the inclusion of participants from the healthy general population without detection bias. In addition, we compared GB diseases with other risk factors for different types of polyps located at different parts of the colon, which was not deeply explored in other studies and has thus far shown limitations in design. We believe this objective design may lower the selection bias of our study. Our data also reported adequate ADR, which is not emphasized or mentioned in other similar studies. We provide this parameter to prove the quality of the study. However, there are also some limitations of our study. First, our study is limited by its retrospective setting. Another limitation is that sessile serrated adenoma prevalence is low in our patient group. Better communication with pathologists may be necessary to explore possible linkage to another pathogenesis. Additionally, other possible confounding factors, such as a personal dietary habit, tobacco use, alcohol consumption, and medication history, including vitamin, NSAIDs, aspirin, and statins, could not be investigated because of incomplete data. Lastly, stool samples were not obtained, and we did not investigate the phenotype of microbiota or explain its association between secondary bile acid and proximal colon polyps. Additional studies are warranted to investigate mechanisms underlying the association between colon polyp development and GB disease, confirming the relationship and clarifying controversial points.

## 5. Conclusions

We suggest that GB polyps are associated with proximal colon polyps. Colonoscopy may be a more effective strategy for screening proximal precancerous lesions among patients with GB polyps. The association between GB disease and colon polyps demands further prospective investigation.

## Figures and Tables

**Table 1:** Comparison of baseline characteristics of study participants.

	All	Any colon polyp			Proximal colon polyp			Distal colon polyp		
	(*N* = 3136)	Any polyp (*N* = 1551)	No any polyp (*N* = 1585)	*P* value	Any proximal polyp (*N* = 480)	No any proximal polyp (*N* = 2656)	*P* value	Any distal polyp (*N* = 1244)	No any distal polyp (*N* = 1892)	*P* value
Mean age ± SD (years)	49.31 ± 11.29	50.42 ± 11.55	48.21 ± 10.92	<0.001	54.05 ± 9.98	48.45 ± 11.3	<0.001	49.55 ± 11.8	49.15 ± 10.95	0.584
Male, *n* (%)	1776 (56.6)	920 (59)	856 (54)	0.003	322 (67)	1454 (55)	<0.001	718 (58)	1058 (56)	0.32
Mean BMI ± SD (kg/m^2^)	24.24 ± 3.54	24.32 ± 3.54	24.15 ± 3.55	0.708	24.22 ± 3.39	24.24 ± 3.57	0.899	24.36 ± 3.57	24.16 ± 3.53	0.152
DM, *n* (%)	143 (4.6)	82 (5)	61 (4)	0.054	26 (5)	117 (4)	0.328	66 (5)	77 (4)	0.105
Mean TG ± SD (mg/dL)	140.31 ± 94.42	143.42 ± 98.76	137.27 ± 89.91	0.068	155.58 ± 121.23	137.55 ± 88.47	<0.001	141.93 ± 101.6	139.25 ± 89.4	0.98
Mean TC ± SD (mg/dL)	197.14 ± 35.75	197.5 ± 36.07	196.79 ± 35.44	0.578	199.16 ± 36.99	196.77 ± 35.52	0.482	197.66 ± 36.34	196.79 ± 35.36	0.546
GB stone, *n* (%)	212 (6.8)	123 (7.9)	89 (5.6)	0.01	43 (9.0)	169 (6.3)	0.037	95 (7.6)	117 (6.2)	0.113
GB polyp, *n* (%)	512 (16.3)	266 (17.2)	246 (15.5)	0.217	102 (21.3)	410 (15.4)	0.002	205 (16.5)	307 (16.2)	0.851
Family history of CRC, *n* (%)	180 (5.7)	105 (7)	76 (5)	0.018	32 (6.6)	149 (5.6)	0.361	86 (7)	94 (5)	0.022

SD: standard deviation; BMI: body mass index; DM: diabetes mellitus; TG: triglycerides; TC: total cholesterol; GB: gallbladder; CRC: colorectal cancer.

**Table 2:** Univariate and multivariate analyses for factors of colon polyp in any colon polyp group.

	Any colon polyp				Any hyperplastic polyp				Any adenomatous polyp			
	Univariate 95% CI of estimated *β*	*P* value	Multivariate 95% CI of estimated *β*	*P* value	Univariate 95% CI of estimated *β*	*P* value	Multivariate 95% CI of estimated *β*	*P* value	Univariate 95% CI of estimated *β*	*P* value	Multivariate 95% CI of estimated *β*	*P* value
Age (years)	1.011-1.024 (1.018)	<0.001	1.010-1.023 (1.016)	<0.001	1.007-1.022 (1.013)	<0.001	1.006-1.021 (1.014)	<0.001	1.019-1.036 (1.028)	<0.001	1.018-1.035 (1.027)	<0.001
Sex (male)	1.078-1.43 (1.242)	0.003	1.064-1.427 (1.232)	0.005	1.046-1.449 (1.231)	0.012	1.057-1.466 (1.245)	0.009	1.135-1.627 (1.359)	0.001	1.114-1.615 (1.341)	0.002
BMI (kg/m^2^)	0.994-1.034 (1.014)	0.17			0.992-1.038 (1.015)	0.208			0.99-1.048 (1.015)	0.25		
DM	0.994-1.957 (1.395)	0.055	0850-1.700 (1.203)	0.297	0.904-1.96 (1.331)	0.148			0.978-2.215 (1.472)	0.064		
TG (mg/dL)	1.00-1.001 (1.001)	0.07	0.999-1.001 (1.000)	0.488	1.00-1.001 (1.00)	0.437			1.00-1.002 (1.001)	0.143		
TC (mg/dL)	0.999-1.003 (1.001)	0.578			1.00-1.002 (1.001)	0.063			0.999-1.004 (1.001)	0.334		
GB stone	1.092-1.92 (1.448)	0.01	0.983-1.744 (1.309)	0.066	1.017-1.93 (1.401)	0.039	0.933-1.790 (1.292)	0.123	1.089-2.150 (1.53)	0.014	0.939-1.879 (1.328)	0.109
GB polyp	0.932-1.362 (1.127)	0.217			0.902-1.392 (1.12)	0.304			0.923-1.477 (1.168)	0.196		
Family history of CRC	1.064-1.954 (1.442)	0.018	1.059-1.954 (1.439)	0.02	1.169-2.274 (1.63)	0.04	1.179-2.202 (1.648)	0.003	0.789-1.734 (1.17)	0.435		

BMI: body mass index; DM: diabetes mellitus; TG: triglycerides; TC: total cholesterol; GB: gallbladder; CRC: colorectal cancer.

**Table 3:** Univariate and multivariate analyses for factors of colon polyp in the proximal colon polyp group.

	Any proximal colon polyp				Any proximal hyperplastic polyp				Any proximal adenomatous polyp			
	Univariate 95% CI of estimated *β*	*P* value	Multivariate 95% CI of estimated *β*	*P* value	Univariate 95% CI of estimated *β*	*P* value	Multivariate 95% CI of estimated *β*	*P* value	Univariate 95% CI of estimated *β*	*P* value	Multivariate 95% CI of estimated *β*	*P* value
Age (years)	2.058-3.109 (2.53)	<0.001	1.037-1.057 (1.047)	<0.001	1.02-1.049 (1.035)	<0.001	1.021-1.051 (1.036)	<0.001	1.041-1.064 (1.053)	<0.001	1.042-1.066 (1.054)	<0.001
Sex (male)	1.372-2.068 (1.685)	<0.001	1.303-1.999 (1.614)	<0.001	1.152-2.246 (1.608)	0.005	1.047-2.078 (1.475)	0.026	1.4-2.288 (1.79)	<0.001	1.354-2.255 (1.747)	<0.001
BMI (kg/m^2^)	0.971-1.026 (0.998)	0.893			0.964-1.053 (1.008)	0.725			0.964-1.029 (0.996)	0.808		
DM	0.803-1.923 (1.243)	0.329			0.449-2.137 (0.98)	0.96			0.815-2.212 (1.343)	0.217		
TG (mg/dL)	1.001-1.003 (1.002)	<0.001	1.001-1.002 (1.001)	0.007	1.001-1.003 (1.002)	0.002	1.001-1.00 (1.002)	0.008	1.000-1.003 (1.002)	0.008	1.000-1.002 (1.001)	0.091
TC (mg/dL)	0999-1.005 (1.002)	0.179			0.997-1.006 (1.001)	0.574			0.999-1.005 (1.002)	0.243		
GB stone	1.021-2.055 (1.448)	0.038	0.816-1.69 (1.174)	0.388	0.787-2.461 (1.392)	0.255			0.993-2.24 (1.491)	0.054	0.767-1.787 (1.171)	0.764
GB polyp	1.16-1.884 (1.478)	0.002	1.108-1.830 (1.424)	0.006	1.065-2.3 (1.565)	0.023	1.031-2.249 (1.523)	0.034	1.062-1.891 (1.417)	0.018	1.003-1.821 (1.351)	0.048
Family history of CRC	0.810-1.784 (1.202)	0.362			0.898-2.821 (1.592)	0.112			0.639-1.709 (1.045)	0.861		

BMI: body mass index; DM: diabetes mellitus; TG: triglycerides; TC: total cholesterol; GB: gallbladder; CRC: colorectal cancer.

**Table 4:** Univariate and multivariate analyses for factors of colon polyp in the distal colon polyp group.

	Any distal colon polyp				Any distal hyperplastic polyp				Any distal adenomatous polyp			
	Univariate 95% CI of estimated *β*	*P* value	Multivariate 95% CI of estimated *β*	*P* value	Univariate 95% CI of estimated *β*	*P* value	Multivariate 95% CI of estimated *β*	*P* value	Univariate 95% CI of estimated *β*	*P* value	Multivariate 95% CI of estimated *β*	*P* value
Age (years)	0.997-1.001 (1.003)	0.328			0.996-1.011 (1.013)	0.393			0.994-1.013 (1.003)	0.496		
Sex (male)	0.931-1.243 (1.076)	0.32			1.152-2.246 (1.608)	0.005	0.931-1.298 (1.099)	0.264	1.152-2.246 (1.608)	0.005		
BMI (kg/m^2^)	0.996-1.037 (1.016)	0.124			0.991-1.038 (1.015)	0.22			0.992-1.038 (1.015)	0.208		
DM	0.943-1.85 (1.321)	0.106			0.891-1.915 (1.306)	0.171			0.791-2.056 (1.276)	0.318		
TG (mg/dL)	1.00-1.001 (1.00)	0.437			1.00-1.001 (1.00)	0.246			0.999-1.001 (1.00)	0.817		
TC (mg/dL)	0.999-1.003 (1.001)	0.504			0.998-1.003 (1.001)	0.643			0.998-1.004 (1.001)	0.446		
GB stone	0.947-1.661 (1.254)	0.114			0.903-1.71 (1.243)	0.183			0.868-1.904 (1.286)	0.21		
GB polyp	0.84-1.236 (1.019)	0.851			0.801-1.25 (1.001)	0.993			0.815-1.410 (1.072)	0.621		
Family history of CRC	1.051-1.921 (1.421)	0.023			1.103-2.148 (1.539)	0.011	1.108-2.15 (1.546)	0.01	0.825-1.97 (1.275)	0.274		

BMI: body mass index; DM: diabetes mellitus; TG: triglycerides; TC: total cholesterol; GB: gallbladder; CRC: colorectal cancer.

## Data Availability

Numerical data is available to interested readers upon request to the corresponding author of this article.
